# Proliferative verrucous leukoplakia; a critical appraisal of the diagnostic criteria

**DOI:** 10.4317/medoral.18912

**Published:** 2013-03-25

**Authors:** Vinicius C. Carrard, Elisabeth R.E.A. Brouns, Isaäc van der Waal

**Affiliations:** 1 VU medical center/ ACTA, Dept. of Oral and Maxillofacial Surgery/Pathology, Amsterdam, the Netherlands; 2 Federal University of Rio Grande do Sul, School of Dentistry, Porte Alegre, Rio Grande do Sul, Brazil; 3 ….

## Abstract

Since its introduction in the literature in 1985, the term proliferative verrucous leukoplakia (PVL) has been the subject of an ongoing discussion with regard to its definition. Widespread or multifocal occurrence of oral leukoplakia is not just synonymous to PVL. In the present treatise the proposal is made to require the involvement of more than two oral oral subsites, a total added seizeof the leukoplakic areas of at least 3 centimeters, and a well documented period of at least five years of disease evolution being characterized by spreading and the occurrence of one or more recurrences in a previously treated area.

** Key words:**Oral premalignant lesions, leukoplakia, verrucous leukoplakia.

## Introduction

The term proliferative verrucous leukoplakia (PVL) has been defined by Hansen et al. (1) as a disease of unknown origin, that clinically often begins as a single white lesion and along time tends to become multifocal, growing slowly and progressively. It should be acknowledged that some lesions may initially be pinkish or even red rather than white. Hansen et al. (1) description of PVL includes the predilection for elderly women, a strong tendency to recur after treatment and, in many cases, inevitably, the development of squamous cell carcinoma. Tobacco habits were quite common in Hansen et al. (1) series. All sites in the oral cavity can be affected, not showing any preference for specific oral subsites.

Since Hansen et al’s publication many cases diagnosed as PVL have been published, questioning the etiology, diagnostic criteria and appropriate treatment. In general, the diagnostic criteria that have been used in the various studies are diverse and many cases actually seem to represent widespread, multifocal leukoplakia. Cerero Lapiedra et al. (2) proposed diagnostic criteria of PVL using a set of major and minor criteria ( Table 1). Some of these criteria are well accepted, but others seem to be somewhat debatable. In this treatise both the major and minor diagnostic criteria will be commented upon.

**Table 1:**
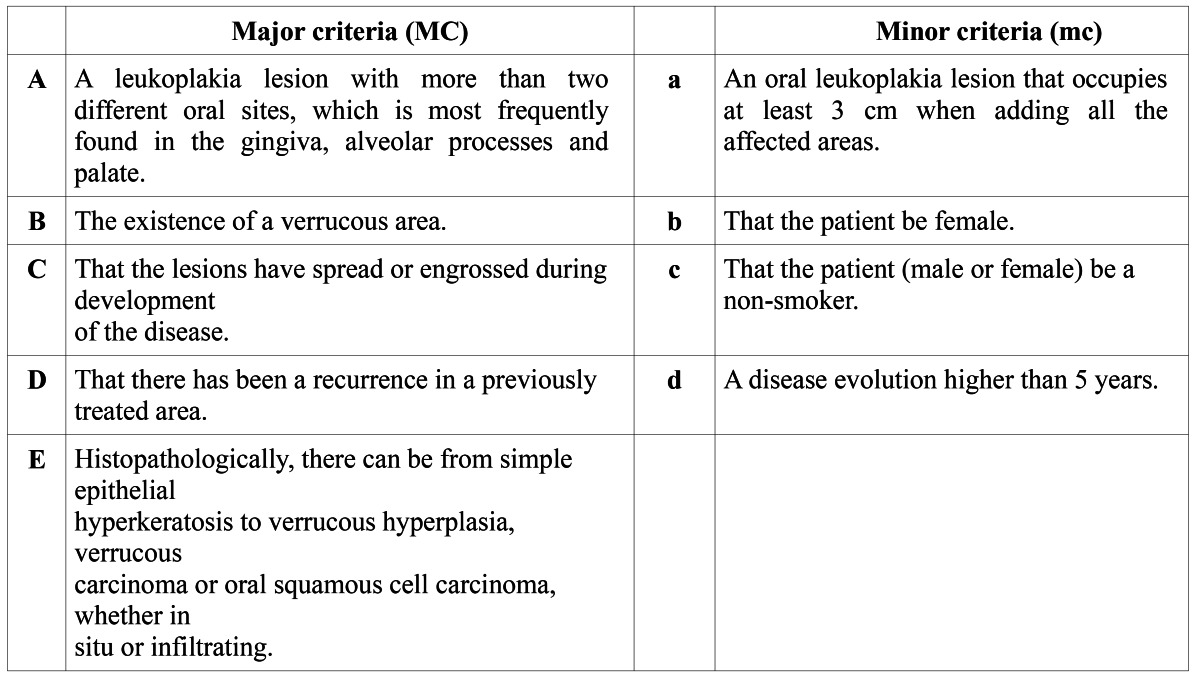
Proposal of major and minor diagnostic criteria for PVL recommended by Cerero-Lapiedra et al. (2010).

Critical appraisal of the diagnostic criteria

In order to make the diagnosis of PVL, Cerero-Lapiedra et al. (2) proposed that one of the two following combinations of criteria mentioned in table 1 should be met:

1. Three major criteria (being E among them) or.

2. Two major criteria (being E among them) + two minor criteria.

2.1 Major criteria

Major criterion A. Cerero-Lapiedra et al. (2) considered involvement of keratinized mucosae (gingiva and palate) as the most frequently involved sites in PVL, although five out of seven case series on which their proposal was based on showed involvement of the buccal mucosa as the first or second most commonly affected site. It should be emphazised, as mentioned by Hansen et al, that any site of the oral cavity may be involved. Therefore, we suggest to limit criterion A to: “Leukoplakia involving more than two oral subsites, either separate or in continuity”. The following oral subsites are recognized: dorsum of the tongue (unilateral or bilateral), border of the tongue, cheek mucosa, alveolar mucosa or gingiva upper jaw, alveolar mucosa or gingiva lower jaw, hard and soft palate, floor of the mouth, upper lip and lower lip.

Major criterion B. The presence of one or more verrucous or wartlike areas is, indeed, a condition sine qua non in the diagnosis of PVL.

Major criterion C. Although spreading and/or enlarging during development is not exclusive for PVL and may occur in any type of leukoplakia, we suggest to maintain this criterion.

Major criterion D. Although recurrence in a previously treated area of leukoplakia is not exclusive for a diagnosis of PVL, it seems appropriate to maintain this criterion.

Major criterion E. Cerero-Lapiedra et al. (2) mentioned that PVL starts as a simple hyperkeratosis, which can progress, with or without stages of various degrees of epithelial dysplasia, to verrucous hyperplasia, verrucous carcinoma or squamous cell carci-noma. However, this observation is not limited to PVL and is, therefore, difficult to accept as a criterion for PVL.

2.2 Minor criteria

Minor criterion a. Many single or multifocal leukoplakias (non-PVL), adding all the affected areas may measure 3 centimeters or more in seize. There seems to be, indeed, some value in requiring a minimum seize for a diagnosis of PVL.

Minor criterion b. Since almost 20% of the cases reviewed by Cerero-Lapiedra et al. (2) including Hansen et al’s paper, were males, female gender should not be included as a (minor) criterion for the diagnosis of PVL.

Minor criterion c. The percentage of smokers in the case series selected by Cerero-Lapiedra et al. (2) varied between 23% and 70%. Therefore, it does not seem justified to include being a non-smoker as a (minor) criterion of PVL.

Minor criterion d. A period of diseases evolution of at least five years, as suggested by Cerero-Lapiedra is more or less in accordance with Hansen et al’s experience.

Conclusion and recommendation

The importance of the diagnosis of PVL lies in the awareness of both the clinician and pathologist that apparently innocent looking oral verrucous lesions, irrespective of their colour and irrespective of the presence of dysplasia may in time progress into verrucous carcinoma or squamous cell carcinoma. In view of our comments on the major and minor diagnostic criteria proposed by Cerero-Lapiedra et al. (2) we suggest to simplify the diagnostic criteria of PVL by omitting the distinction between major and minor criteria as is shown in  Table 2

**Table 2:**
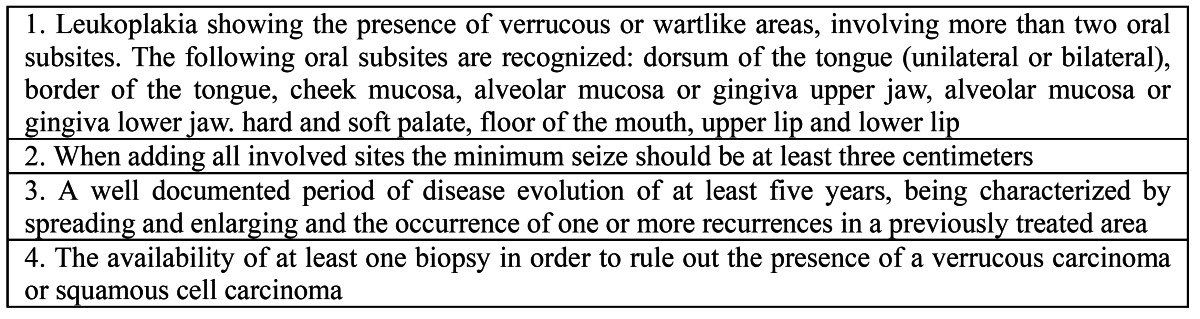
Modified diagnostic criteria for PVL; all four criteria should be met.
